# Physiological predictors of cardiorespiratory fitness in children and adolescents with cystic fibrosis without ventilatory limitation

**DOI:** 10.1177/17534666211070143

**Published:** 2022-01-10

**Authors:** Marcella Burghard, Tim Takken, Merel M. Nap-van der Vlist, Sanne L. Nijhof, C. Kors van der Ent, Harry G.M. Heijerman, H.J. Erik Hulzebos

**Affiliations:** Child Development, Exercise, and Physical Literacy Center, Wilhelmina Children’s Hospital, University Medical Center Utrecht, P.O. Box 85090, 3508 EA Utrecht, The Netherlands; Cystic Fibrosis Center Utrecht, University Medical Center Utrecht, Utrecht, The Netherlands; Child Development, Exercise, and Physical Literacy Center, Wilhelmina Children’s Hospital, University Medical Center Utrecht, Utrecht, The Netherlands; Department of Social Pediatrics, Wilhelmina Children’s Hospital, University Medical Center Utrecht, Utrecht, The Netherlands; Department of Social Pediatrics, Wilhelmina Children’s Hospital, University Medical Center Utrecht, Utrecht, The Netherlands; Cystic Fibrosis Center Utrecht, University Medical Center Utrecht, Utrecht, The Netherlands; Department of Pediatric Pulmonology, Wilhelmina Children’s Hospital, University Medical Center Utrecht, Utrecht, The Netherlands; Cystic Fibrosis Center Utrecht, University Medical Center Utrecht, Utrecht, The Netherlands; Division Heart and Lung, Department of Pulmonology, Cystic Fibrosis Center Utrecht, University Medical Center Utrecht, Utrecht, The Netherlands; Child Development, Exercise, and Physical Literacy Center, Wilhelmina Children’s Hospital, University Medical Center Utrecht, Utrecht, The Netherlands; Cystic Fibrosis Center Utrecht, University Medical Center Utrecht, Utrecht, The Netherlands

**Keywords:** cardiorespiratory fitness, cystic fibrosis, glucose tolerance, *Pseudomonas Aeruginosa*

## Abstract

**Objectives:**

[1] To investigate the cardiorespiratory fitness (CRF) levels in children and adolescents with cystic fibrosis (CF) with no ventilatory limitation (ventilatory reserve ⩾ 15%) during exercise, and [2] to assess which physiological factors are related to CRF.

**Methods:**

A cross-sectional study design was used in 8- to 18-year-old children and adolescents with CF. Cardiopulmonary exercise testing was used to determine peak oxygen uptake normalized to body weight as a measure of CRF. Patients were defined as having ‘low CRF’ when CRF was less than 82%predicted. Physiological predictors used in this study were body mass index z-score, *P. Aeruginosa* lung infection, impaired glucose tolerance (IGT) including CF-related diabetes, CF-related liver disease, sweat chloride concentration, and self-reported physical activity. Backward likelihood ratio (LR) logistic regression analysis was used.

**Results:**

Sixty children and adolescents (51.7% boys) with a median age of 15.3 years (25th–75th percentile: 12.9–17.0 years) and a mean percentage predicted forced expiratory volume in 1 second of 88.5% (±16.9) participated. Mean percentage predicted CRF (ppVO_2peak/kg_) was 81.4% (±12.4, range: 51%–105%). Thirty-three patients (55.0%) were classified as having ‘low CRF’. The final model that best predicted low CRF included IGT (*p* = 0.085; Exp(B) = 6.770) and *P. Aeruginosa* lung infection (p = 0.095; Exp(B) = 3.945). This model was able to explain between 26.7% and 35.6% of variance.

**Conclusions:**

CRF is reduced in over half of children and adolescents with CF with normal ventilatory reserve. Glucose intolerance and *P. Aeruginosa* lung infection seem to be associated to low CRF in children and adolescents with CF.

## Introduction

Cystic fibrosis (CF) is a heritable disease characterized by dysfunction of the CF transmembrane conductance regulator (CFTR) epithelial chloride channel. This leads to dehydration and thickening of secretions in the airways, pancreatic ducts, and other body parts, resulting in progressive obstruction, inflammation, and recurrent infections of the airways and several other organs.^1^ In the past 10 years, treatment and control of CF has improved drastically, and the average survival of people with CF (pwCF) has significantly increased.^1^ Peak oxygen uptake (VO_2peak_), a measure of cardiorespiratory fitness (CRF), is associated with quality of life^2^ and survival^3^ in pwCF. Indeed, Nixon *et al.*^4^ reported, that pwCF with a VO_2peak_ ⩾ 82%predicted have a survival rate of 83% at 8 years, as compared with rates of 51% and 28% for pwCF with middle (59%–81%predicted) and lowest levels (⩽58%predicted) of CRF. Similar findings were reported in a large international study by Hebestreit *et al.*^3^ in which it was demonstrated that children and adults with CF with the highest VO_2peak_, for example, ⩾82%predicted, had a 72% lower risk of dying and 49% lower risk of receiving a lung transplant over a 10-year period compared with pwCF in the middle and lowest VO_2peak_ groups. In addition, in the Statement of Exercise Testing in CF, it was stated that CRF should be classified as abnormal when CRF is less than 82%predicted of VO_2peak_. Taken together, a cutoff point of 82% for CRF seems to be of clinical value in this population.

Given the strong link between survival and CRF in pwCF, identifying predictors of CRF is critical for supporting optimal health in this vulnerable population. It is acknowledged that CRF is (largely) dependent on pulmonary function, especially forced expiratory volume in one second (FEV_1_), in both children and adults with CF.^5–8^ Studies have shown that physical activity and CRF are positively related^9–11^ in pwCF, and as it is acknowledged that both CRF^9,12^ and pulmonary function^9^ decline with age, it is relevant to uncover factors in a pediatric population with little pulmonary loss. To date, much of our understanding of the correlates of CRF in pwCF comes from populations with reduced pulmonary function or those with ventilatory limitation.^10,13,14^ The physiological factors related to CRF in pwCF with normal pulmonary function and no ventilatory limitation are less well known. Establishing predictors of CRF in pediatric patients with CF and no ventilatory limitations will allow for optimal monitoring and treatment early in CF management, resulting in the best possible outcome for pwCF across the lifespan. Growing evidences emerges that skeletal muscle dysfunction or abnormality is potentially related to lower CRF levels. Causes for dysfunctional skeletal muscles, including atrophy and weakness, include a variety of factors such as inflammation, hypoxemia, oxidative stress, exacerbations, and use of corticosteroids.^15^ However, in mild-to-moderate severe disease status, these factors are less likely to result in major skeletal muscle problems. But, muscle abnormalities were still present in female athletes with normal pulmonary function, who had higher physical activity levels measured with accelerometer and self-report diaries, compared to their healthy training partners, ^16^ suggesting factors directly related to CF pathology. Since CFTR is expressed in skeletal muscle it is plausible that CFTR dysfunction affects skeletal muscle function and consequently, affects CRF directly. Although CFTR genotype was not associated with CRF^13^ in a large, international multicenter study, no studies have investigated the link between CRF and CFTR function, defined using a proxy measure of sweat chloride concentration. Additional plausible correlates of CRF include nutritional status (body mass index, BMI), CF-related diabetes (CFRD), CF-related liver disease (CFRLD), and chronic infection with *P*. A*eruginosa*, which are all known co-morbidities and established predictors for survival in CF patients.^3,9^ In the studies of Foster *et al.*^14^ and Causer *et al.*^17^ lower CRF levels were found for patients with impaired glucose tolerance (IGT) and CFRD compared to patients with normal glucose tolerance. However, variables as sex and age were included in their models, which are already known covariates for CRF in CF. Specific exclusion of ventilatory limited patients and inclusion of *P. Aeruginosa* and CFTR function have not yet been analyzed all together. Physical activity has also been linked with a slower rate of decline in pulmonary function in pediatric patients with CF.^18^ While some of these factors have been independently associated with CRF^9–11^ in pwCF, no study to date has examined their combined association with CRF. In addition, when physiological predictors are detected, possible prevention and treatment can occur early in CF management, most likely to result in highly improved outcomes in lifespan CF care. Therefore, we aimed to (1) investigate the CRF levels in pediatric patients with CF without ventilatory limitation during exercise, and (2) determine the relationship between physiological factors (BMI *z*-score, glucose tolerance status, presence of CFRLD, colonization of *P. Aeruginosa*, sweat chloride concentration, and self-reported physical activity) and CRF in these patients.

## Materials and methods

### Subjects

We used cross-sectional data from children and adolescents with CF between 8 and 18 years of age collected in the Wilhelmina Children’s Hospital, University Medical Center Utrecht, from December 2016 through January 2019. Only those with no ventilatory limitation during exercise (described below) were included in this study, and all patients were free from acute pulmonary exacerbation at the time of testing. All patients have signed informed consent for use of standard-care data for scientific research purposes. These data include all demographic and clinical data from routine outpatient visits, which could be collected from the electronic patient records, as outlined below. This cross-sectional study was part of the larger PROactive cohort study with annual measurements with chronically ill patients and their families,^19^ including registration for a web-based tool to fill in questionnaires. This PROActive cohort study was classified by the institutional review board as exempt based on the Medical Research Involving Human Subjects Act (METC number: 16-707/C).

#### Study procedures

All patients of the outpatient clinic and their parents were approached by email 3 weeks before their regular, annual CF check-up. They were invited to register for the study at home using a web-based tool (www.hetklikt.nu). Patients were asked to complete the electronic questionnaire through the web portal before their outpatient clinic visit. Patients who did not respond to the original invitation received a single reminder email and reminder telephone call was used.

#### Cardiorespiratory fitness

CRF was assessed using a cardiopulmonary exercise test (CPET) according to the Godfrey protocol on an electronically braked cycle ergometer (Lode Corrival, Lode BV, Groningen, the Netherlands).^20^ During the test, subjects breathed through an air-tight face mask (Hans Rudolph Inc., Shawnee, USA), connected to a calibrated metabolic cart (Geratherm, Bad Kissingen, Germany). Objective criteria to assess the quality of the delivered effort were (1) peak heart rate > 95%predicted and (2) respiratory exchange ratio > 1.00.^21^ Patients had to meet at least one out of two objective criteria for the test to be considered of maximal effort. Peak oxygen uptake adjusted for body weight (VO_2peak/kg_) was used as primary outcome measurement for CRF. Because scaling of exercise variables is important in growing children, and both age and sex^22–24^ are related to CRF, we only included the predicted value for VO_2peak/kg_ in the statistical analyses. Reference equations from Bongers *et al.*^24^ were used to calculate percentage predicted values for VO_2peak/kg_. Patients were classified as having ‘low CRF’ when CRF <82%predicted of VO_2peak/kg_, based on the criteria from Hebestreit *et al.*^3,21^ Patients with a ventilatory limitation during maximal exercise were excluded based on ventilatory reserve (VR), which was calculated as VR = 100 − ((VE_peak_/MVV) • 100), where VE_peak_ is the maximal volume of air exhaled per minute at peak exercise and MVV is the estimated maximal voluntary ventilation (MVV = FEV_1_ × 35; in children).^25^ Those with a VR < 15% were classified as having ventilatory limitation, based on the ATS/ACCP statement on CPET,^26^ and excluded from subsequent analyses.

#### Clinical assessments

Clinical parameters including mutation, glucose tolerance status including IGT and CF-related diabetes, CFRLD, colonization with *P. Aeruginosa*, sweat chloride concentration, and use of CFTR-modulating therapies were extracted from patient electronic medical record from the date closest to the date of CPET completion. In our center, routine screening for CFRD and CFRLD was performed according to the European CF Society Standards of Care. Regarding CFRLD, this includes periodic liver enzyme testing, and when indicated ultrasonography and liver biopsies. CFRLD includes cirrhosis with and without hypertension and/or hypersplenism. For diagnosing CFRD, annual oral glucose testing is performed.^27^ Being colonized with *P. Aeruginosa* was defined as having of two or more positive cultures in the last year. To gain more insight in disease status, number of intravenous treatments in the last year was searched in the medical records as well. Parameters were searched up to 18 months before completing of the CPET.

#### BMI (*z*-score)

Non-fasting measures of body weight and height were collected using a calibrated electronic scale (Seca, Hamburg, Germany) and stadiometer (Ulmer Stadiometer, Ulm, Germany), respectively. The patients were dressed in lightweight clothing and removed any footwear during the measurement. BMI was calculated by dividing body weight in kilograms by height in meters squared. *Z*-scores for BMI were calculated based on the fifth national growth study of TNO (2010).^28^

#### Pulmonary function

Forced expiratory volume in one second (FEV_1_) was measured with spirometry and expressed as a percentage of predicted (ppFEV_1_). Spirometry was performed according to the ERS/ATS recommendations using the Spirostik system (Geratherm, Bad Kissingen, Germany). The Global Lung Initiative (GLI) lung function reference equations were used for the calculation of ppFEV_1_.^29^

#### Self-reported physical activity

Participants were asked: ‘Over the past 7 days, on how many days were you physically active for a total of at least 60 minutes per day?’. Response options included: 0 days, 1 day, 2 days, 3 days, 4 days, 5 days, 6 days, and 7 days. This question was derived from the Health Behavior in School-aged children (HBSC*)* study, an international school-based survey with data collected through self-report.^30^ The reported number of days was included in the statistical analysis.

### Statistical analysis

Descriptive statistics were used to summarize demographic, clinical, and CRF characteristics. Distribution of the data was assessed with Q-Q plots and histograms and values are reported with the mean ± SD in case of normal distributed data, and with median ± 25th and 75th percentiles in case of non-normal distributed data.

Multivariable binary logistic regression analysis was applied, including level of fitness (low CRF *versus* normal CRF) as dependent variable. The backward likelihood ratio method was applied, with 0.05 as entry for probability stepwise and 0.10 for removal. BMI *Z*-score, sweat chloride concentration, and self-reported physical activity levels were included as continuous variables. Glucose tolerance status, CFRLD, and colonization with *P. Aeruginosa* were included as categorical variables, in which 1 coded for presence and 0 coded for absence of the co-morbidities.

Confounders for CRF including age, sex,^22–24^ and body mass,^24,31^ were integrated into our outcome of percentage predicted VO_2peak/kg_. Analysis was performed using SPSS version 25.0 (IBM Corp, Armonk, USA), and differences with a *p* value < 0.05 were considered statistically significant.

## Results

### Characteristics of the study population

A total of 115 children and adolescents were invited to participate in this study. Of these, 72 responded (response rate of 62.6%) and data could only be used from 68 patients. Three patients did not provide consent for use of data from their clinic medical record, and one patient refused to wear a facemask during the exercise test. Reasons for not participating were time investment, filling out too many questionnaires, and personal circumstances. Of the 68 participants, eight patients had a VR of less than 15% during cardiopulmonary exercise testing, and were excluded for data analysis. Therefore, 60 patients with CF (51.7% male) were included in our analyses. Demographic and clinical characteristics are presented in Table 1. IGT including CFRD was diagnosed at a mean age of 12.5 ± 2.7 years, and CFRLD was diagnosed at a mean age of 8.3 ± 3.1 years. Time from diagnosis of IGT including CFRD and performing CPET was mean 2.6 ± 2.5 years, and median 5.0 (3.0–7.0 25th–75th percentile) years for CFRLD. A total of 10 patients (16.7%) used CFTR modulating therapies, at time of data collection. Mean time on CFTR modulating therapies was 15.4 ± 9.0 months (range 1–28 months). Data for sweat chloride concentration were missing for 11 patients (18%).

**Table 1.** Patient characteristics, cardiorespiratory fitness, and self-reported physical activity of all patients with CF (*n* = 60) and divided by level of fitness: normal CRF (*n* = 27) and low CRF, in children and adolescents (*n* = 33).

	Total patients (*n* = 60)	Normal CRF (ppVO_2peak_kg_ ⩾ 82%) (*n* = 27)	Low CRF (ppVO_2peak_kg_ < 82%) (*n* = 33)
Patient characteristics			
Age (years) (median, 25th–75th percentile)	15.3 (12.9–17.0)	13.8 (10.0–16.7)	15.8 (13.8–17.7)
Sex (% boys)	31 (51.7)	16 (59.3)	15 (45.5)
BMI (*z*-score) (median, 25th–75th percentile)	−.3 (–.9 to .4)	−.3 (–.8 to .3)	−.3 (–1.0 to .5)
Pulmonary function			
ppFEV_1_ (%)	88.5 (16.9)	90.9 (14.4)	86.5 (18.6)
Mutation			
Homozygous F508del (*n*, %)	34 (56.7)	14 (51.9)	20 (60.6)
Heterozygous F508del (*n*, %)	25 (41.7)	13 (48.2)	12 (36.4)
Other (*n*, %)	1 (1.7)	0 (0.0)	1 (3.0)
CF specific			
Sweat chloride concentration (mmol/L)	99.1 (15.0)	95.3 (13.7)	102.5 (15.5)
CFTR modulating therapies; yes (*n*, %)	10 (16.7)	3 (11.1)	7 (21.2)
IV treatment; yes (*n*, %)	14 (23.3)	5 (18.5)	9 (27.3)
Comorbidities			
IGT, including CFRD (*n*, %)	16 (26.7)	4 (14.8)	12 (36.4)
CFRLD (*n*, %)	22 (36.7)	13 (48.1)	9 (27.3)
Colonization *P. Aeruginosa* (*n*, %)	20 (33.9)	7 (25.9)	13 (40.6)
Cardiorespiratory fitness and exercise response			
ppVO_2peak/kg_ (%)	81.4 (12.4)	92.6 (6.6)	72.3 (7.5)
Physical activity behavior			
Physical activity (days) (median, 25th–75th percentile)	4.0 (3.0–7.0)	5.5 (3.0–7.0)	4.0 (3.0–6.0)

BMI (*z*-score), body mass index *z*-score; CF, cystic fibrosis; CFRD, cystic fibrosis–related diabetes; CFRLD, cystic fibrosis–related liver disease; CFTR, CF transmembrane conductance regulator; CRF, cardiorespiratory fitness; IGT, impaired glucose tolerance; IV treatment, number of intravenous treatments in the last year; *P. Aeruginosa, Pseudomonas Aeruginosa*; ppFEV_1_, forced expiratory volume in one second, in percentage predicted; ppVO_2peak/kg_, peak oxygen uptake, related to body weight, in percentage predicted; VO_2_peak, peak oxygen uptake.

### Cardiorespiratory fitness

Mean CRF (ppVO_2peak/kg_) was 81.4% (±12.4, range: 51%–105%). When classified by fitness level, 33 of the patients (55.0%) were identified as having ‘low CRF’ (VO_2peak/kg_ < 82% of predicted) (Table 1).

### Multivariate analysis

The final model included three physiological factors: impaired glucose intolerance and colonization with *P. Aeruginosa* were associated with increased odds of having ‘low CRF’ (odds ratio (OR): 6.770 and 3.945, respectively), while having CFRLD was associated with decreased odds of having ‘low CRF’ deconditioned (OR = .095). Assumptions for linearity and multi-collinearity were satisfied. The final model fit the data adequately (Hosmer and Lemeshow’s *X*^2^ = 1.244, *p* = 0.871), and was able to predict having ‘low CRF’ (Omnibus *X*^2^ (3) = 14.595, *p* = 0.002). The model was able to explain between 26.7% and 35.6% of the variance in fitness level (Table 2)

**Table 2.** Multivariate analysis of level of fitness (low CRF = 1) and possible contributing factors (low CRF based on ppVO_2peak_kg_ < 82%).

Variable	Cox & Snell *R*^2^	Nagelkerke *R*^2^	HL *X*^2^	Sig	B	Wald2	*df*	*p*	Exp(B)	95% CI for EXP (B)
Model	.267	.356	1.244	.871	–	–	–	–	–	–
IGT incl. CFRD (yes/no)	–	–	–	–	1.912	2.961	1	0.085	6.770	0.766–59.794
CFRLD (yes/no)	–	–	–	–	−2.350	6.808	1	0.009	0.095	0.016–0.557
Colonization *P. Aeruginosa* (yes/no)	–	–	–	–	1.373	2.790	1	0.095	3.945	0.788–19.749
Constant	–	–	–	–	0.122	0.082	1	0.775	1.130	–

CFRD, cystic fibrosis–related diabetes; CFRLD, cystic fibrosis–related liver disease; CI, confidence interval; CRF, cardiorespiratory fitness; IGT, impaired glucose tolerance; *P. Aeruginosa, Pseudomonas Aeruginosa.*

## Discussion

Our study showed that 55% of our pediatric sample with CF without ventilatory limitation was classified as having ‘low CRF’. This is much higher than the 9% of healthy peers classified having ‘low CRF’.^24^ This is especially alarming in light of the fact that CRF is associated with survival in pwCF.^3^ Our findings are consistent with other studies that reported lower CRF levels in children and adolescents with CF compared to healthy peers.^32–36^ Indeed, our CRF levels (VO_2peak/kg_) were quite similar to those reported in the literature.^32,33,35,37^ Furthermore, our findings are in line with the study of Foster *et al.*^14^ as well, in which more pwCF with IGT (32%) and CFRD (48%) could be classified as having low CRF, compared to pwCF with normal glucose tolerance (20%).

Our results showed an association between glucose intolerance and low CRF. In literature, the relation of glucose (in)tolerance, and CRF remains unambiguous. Ziegler *et al.*^38^ did not find relevant differences on 6-minute walk test (6mwt) outcome between pwCF with and without IGT. However, the submaximal character of the 6mwt could be a possible explanation for these reported differences. Furthermore, Causer *et al.*^17^ indicated that CRF was reduced in adults with CFRD and IGT but that it is probably related to lung disease severity. The finding of Foster *et al.*^14^ that insulin and glucose values at 120 min during an OGTT were related with CRF in pwCF with normal glucose tolerance, is in line with our results. To state anything about causality is not possible due to our cross-sectional study design, but our results reinforce the proposed relation between glucose tolerance and CRF. Our finding supports the need to screen and act swiftly when children and adolescents with CF show IGT, as this may impact their CRF, and eventually survival. This is particularly important for older individuals with CF where CFRD is more common.^39^ Insulin is known to enhance muscle protein synthesis and inhibit muscle protein breakdown,^40,41^ and both insulin resistance and insulin deficiency lead to a reduction of insulin signaling in the skeletal muscle.^41–43^ Skeletal muscle is one of the major target organs of insulin and accounts for approximately 75.0% of whole-body insulin-stimulated glucose uptake.^41,44^ Accordingly, reports of abnormal muscle protein metabolism^41–43^ and reduced skeletal muscle mass^41,45,46^ in the patients with IGT and CFRD may be among the factors contributing to reduced CRF levels. However, it is also known that in patients with insulin resistance or diabetes mellitus II, exercise training improved insulin signaling by enhancing microvascular responses to insulin^47–49^ and augmenting capillary blood flow,^49–52^ both of which augmented perfusion and increased glucose and insulin delivery in skeletal muscle.^49,53^ Future studies are needed to establish a causal link between CRF and IGT as well as to explore exercise training modalities.

Another relevant factor in CRF seems colonization with *P. Aeruginosa*. Interestingly, in the population of Ziegler *et al.*,^54^ the prevalence of *P. Aeruginosa* colonization was higher in the patients with oxygen desaturation and lower distance on the 6mwt. Furthermore, the role of *P. Aeruginosa* in altering CRF was previously described by van de Weert-van Leeuwen *et al.*^12^ They found an annual decline of 4.6% in CRF independent of age, pulmonary function, and BMI, when patients were colonized with *P. Aeruginosa*. They suggested that the chronic inflammation seen with *P. Aeruginosa* has negative effects on skeletal muscle.^12,55^ In addition, chronic infection and inflammation may lead to increased intravenous treatment and hospitalization, which negatively affect physical activity, and in turn, CRF.^12^ Our data are consistent with this hypothesis because we observed a higher number of intravenous (IV) treatments in the patients with ‘low CRF’ (Table 1), and the physical activity levels tend to be lower in the ‘low CRF’ group (Table 1), though this was not statistically significant (Supplementary Table). Moreover, murine study results suggest a relation between *P. Aeruginosa* and insulin metabolism as it was found that acute *P. Aeruginosa* colonization induced insulin resistance.^56^ Poorer neuromuscular skills, especially during adolescence, as reported by Gruber *et al.*^57^ could be another factor to take into consideration for lower CRF and physical activity levels, especially engaging in higher intensities. Despite general nutritional improvements in CF care, still growth retardation,^58,59^ delayed puberty,^59,60^ and disturbed body composition^59,61^ are reported, and all could contribute to underdevelopment of the neuromuscular skills and consecutively CRF. Sweat chloride concentration was not related to CRF levels in our sample, and is also consistent with earlier findings that CFTR genotype was not associated with CRF.^13^

Noteworthy is our finding that having CFRLD is associated with lower odds of being classified as having ‘low CRF’. A possible explanation could be related to our finding of a higher prevalence of CFRLD in boys (*n* = 14; 45.2%) compared to girls (*n* = 11, 37.9%), which is consistent with the CF literature.^62^ In our sample, we also found that fewer boys were classified as having ‘low CRF’ (*n* = 15, 48.4%) compared to the girls (*n* = 18, 62.1%) and their CRF levels were significantly higher (mean CRF in boys 84.7% ± 10.6 *versus* 77.9% ± 13.3 in girls, *p* = 0.032). In the general population, girls have already a lower CRF,^22–24^ thus girls with CF may be even more prone to present with lower CRF levels. Still, this relationship between CFRLD and CRF remains ambiguous. Although we included a variety of liver disease severities in our sample, we could not compare by severity due to sample size. Future studies should explore the interaction between sex, CFRLD (and severity), and CRF to provide more insight into our finding. To the best of our knowledge, no other studies have investigated the link between CRF and CFRLD in CF; however, in non-CF populations, most studies suggest that liver disease is associated with reduced CRF.^63–66^

Our findings should be interpreted with some limitations in mind. First, we did not include device-measured physical activity data or data of body composition (fat free mass) because these were not part of our standard CF care. Subjective measures of physical activity are often prone to reporting bias (over or underestimation), thus using for instance accelerometry would most likely provide a more accurate status of a patient’s physical activity level. Data of body composition would facilitate our understanding about possible skeletal muscle (dys)function. This would provide a more in-depth analysis of the relations with (the development) of CRF. However, challenges remain in choosing the best devices, both for physical activity and body composition, as a consequence of rapid development of devices and absence or developing cut-off points and reference values specifically for the CF population. Our sample included a small sample of patients with CFTR modulating therapies which makes it difficult to draw definitive conclusions on their association with CRF. However, this smaller sample corresponds to trends in CF management during our study period. Nonetheless, we did not find any relevant differences for CRF levels between the patients with and without CFTR modulating therapies. Owing to the cross-sectional nature of our study, we were unable to establish causal relationships. Nevertheless, our results indicate that it is important to assess CRF early in life and to have a more holistic view of the physical capacity of the patients in pediatric CF care. Early diagnostics and prevention of CFRD and *P. Aeruginosa* colonization are not only important for maintenance of pulmonary function in the long-term but also for the preservation of CRF. In addition, physical activity promotion and exercise are important clinical aspects in the treatment of CF, but for patients with impaired CRF the prevention of co-morbidities is crucial. The importance of a personalized treatment should be acknowledged. Together with the patient, one should aim for maintaining or optimizing CRF levels. The optimal exercise prescription for patients with CF and glucose intolerance/CFRD is not yet established; however, exercise training interventions have shown to be effective in improving CRF.^67^

In addition to the physiological factors considered in this study, there are several additional factors that may explain the variance in CRF. Psychosocial factors, such as, physical self-efficacy, body image, and self-reported fatigue might be such factors. Embarrassment and non-normality are the two most common barriers to physical activity.^59,68^ Combined with possibly poorer neuromuscular skills, CRF could be affected as well. Self-reported fatigue is more prevalent in children and adolescents with CF compared to their healthy counterparts;^19^ however, causality is extremely difficult with regard to fatigue and was beyond the scope of our study as much was unknown in the relation of physiological predictors and CRF. Future studies should elaborate on these possible other (psychosocial) factors. In addition, future studies should include larger samples with longitudinal data, including sex differences, a variety of CFRLD, device-based measures of physical activity, and assessment of skeletal muscle metabolism. Building on our findings, future research on the optimal treatment and intervention to prevent/increase CRF, improve glucose tolerance, and treat *P. Aeruginosa* infections in pediatric pwCF are warranted.

## Conclusion

Over half of children and adolescents with CF with normal ventilatory reserve present with reduced CRF. IGT and *P. Aeruginosa* infections may be important physiological predictors of CRF in children and adolescents with CF. The possible link between CFRLD and CRF in pwCF necessitates further attention.

## Supplemental Material

sj-docx-1-tar-10.1177_17534666211070143 – Supplemental material for Physiological predictors of cardiorespiratory fitness in children and adolescents with cystic fibrosis without ventilatory limitation Click here for additional data file. Supplemental material, sj-docx-1-tar-10.1177_17534666211070143 for Physiological predictors of cardiorespiratory fitness in children and adolescents with cystic fibrosis without ventilatory limitation by Marcella Burghard, Tim Takken, Merel M. Nap-van der Vlist, Sanne L. Nijhof, C. Kors van der Ent, Harry G.M. Heijerman and H.J. Erik Hulzebos in Therapeutic Advances in Respiratory Disease
