# Supplementary material for: Physiological predictors of cardiorespiratory fitness in children and adolescents with cystic fibrosis without ventilatory limitation
Source: Ther Adv Respir Dis. 2022 Jan 10;16:17534666211070143. doi: 10.1177/17534666211070143 (PMC8755930; doi:10.1177/17534666211070143)
Supplement: sj-docx-1-tar-10.1177_17534666211070143 – Supplemental material for Physiological predictors of cardiorespiratory fitness in children and adolescents with cystic fibrosis without ventilatory limitation [file sj-docx-1-tar-10.1177_17534666211070143.docx]

| **SUPPLEMENTARY TABLE:** RESULT OF BETWEEN GROUP (‘NORMAL CRF’ AND ‘ LOW CRF’ PEDIATRIC PATIENTS WITH CF) TESTING USING MANN-WHITNEY U | | | |
| --- | --- | --- | --- |
|  | **Normal CRF** (ppVO_2peak_kg_≥ 82%)  (n=27) | **Low CRF** (ppVO_2peak_kg_ < 82%)  (n=33) | ***p-value***  (two-tailed) |
| ***Physical activity behavior*** |  |  |  |
| **Physical activity** (days) (median, 25^th^- 75^th^ percentile) | 5.5 (3.0-7.0) | 4.0 (3.0-6.0) | 0.092 |
